# Overexpression of Isoprene Synthase Affects ABA- and Drought-Related Gene Expression and Enhances Tolerance to Abiotic Stress

**DOI:** 10.3390/ijms21124276

**Published:** 2020-06-16

**Authors:** Jia Xu, Livio Trainotti, Mingai Li, Claudio Varotto

**Affiliations:** 1 Department of Biodiversity and Molecular Ecology, Fondazione Edmund Mach, Research and Innovation Centre, via Mach 1, 38010 San Michele all’Adige (TN), Italy; alessia.xu.jia@gmail.com; 2 Dipartimento di Biologia, Università degli Studi di Padova, viale Giuseppe Colombo, 3, 35131 Padova, Italy; livio.trainotti@unipd.it

**Keywords:** isoprene, abscisic acid (ABA), gene transcription, water limitation stress, isoprene synthase, transgenic arabidopsis

## Abstract

Isoprene is the most abundant single biogenic volatile compound emitted by plants. Despite the relevance of this molecule to plant abiotic resistance and its impact on global atmospheric chemistry, little is known about the details of its mechanism of action. Here, we characterized through both physiological and molecular methods the mechanisms of action of isoprene using model transgenic arabidopsis lines overexpressing a monocot isoprene synthase gene. Our results demonstrated the effect that isoprene had on ABA signaling at different tissue-specific, spatial, and temporal scales. In particular, we found that isoprene enhanced stomatal sensitivity to ABA through upregulation of *RD29B* signaling gene. By contrast, isoprene decreased sensitivity to ABA in germinating seeds and roots, suggesting tissue-specific mechanisms of action. In leaves, isoprene caused the downregulation of *COR15A* and *P5CS* genes, suggesting that the enhanced tolerance to water-deprivation stress observed in isoprene-emitting plants may be mediated chiefly by an enhanced membrane integrity and tolerance to osmotic stress.

## 1. Introduction

Plants, as sessile organisms, have developed diverse defense mechanisms to allow them to adapt to sudden and severe environmental changes. Among these, drought (water deficiency) and heat (high temperature) are considered the most detrimental abiotic stresses, which cause severe damage to plant survival and crop production. It is also expected that if drought occurs under the condition of raised temperatures from global warming, water scarcity will set in more severely and rapidly [1], which would result in a greater challenge for plant fitness and productivity.

It has been reported that isoprene (2-methyl-1,3-butadiene, a biogenic volatile compound) plays fundamental roles in protecting plants against oxidative stress under various environmentally unfavorable conditions [2]. Recently, it was estimated that around 70% of non-methane biogenic volatile compounds in the atmosphere annually are derived from isoprene emission alone at the global level [3]. Isoprene’s vast dominating quantities and rapid oxidation reactions can strongly influence atmosphere chemistry and air quality [4]. Isoprene biosynthesis is widespread among perennial and deciduous species [5]. In plants, its emission is further stimulated when leaves are subjected to or recovering from environmental stresses [6,7,8,9]. These findings suggest that isoprene emission is capable of responding to many stresses and provides benefits to plants [5,10]. Hypotheses about the protective mechanisms of isoprene emission to improve plant tolerance against biotic and abiotic stresses include membrane stabilization [11,12], direct reactions with reactive oxygen and nitrogen species [13,14,15], and indirect alteration of ROS signaling [2,16]. Therefore, Vickers and colleagues proposed a unified mechanism by which the antioxidant behavior of isoprene improves a variety of abiotic stresses resistance in isoprene emitting plants [10].

Isoprene synthase (IspS) plays a crucial role in the synthesis of isoprene, the production of which is catalyzed from the dimethylallyl diphosphate (DMADP) produced through the methylerythritol 4-phosphate (MEP) pathway [7]. IspS is the first and only committed enzyme of this side-branch of the major terpene biosynthetic pathway [7]. Therefore, the sole presence of this chloroplast-localized enzyme is sufficient to cause a non-emitting plant to emit isoprene (reviewed in Reference [17]). Since the first isolation of an isoprene synthase sequence more than two decades ago [18], several functional studies based on the overexpression or downregulation of *IspS* genes from different species have exploited this peculiarity of *IspS* to engineer isoprene emission into natural non-emitters or to eliminate it from natural emitters (reviewed in Reference [19]). This, in turn, provided ample opportunities to study in plant species from different families (e.g., tobacco, poplar and Arabidopsis) the physiological mechanisms by which isoprene emission enhances tolerance under heat stress [20,21,22,23]. By contrast, the positive effects of isoprene emission in photosynthetic and whole-plant drought responses in transgenic plants were just demonstrated a few years ago [24,25]. These previous studies provided evidence that isoprene emission suppressed the increase of reactive oxygen species (ROS) content, thus protecting the photosynthetic apparatus under drought stress and during the recovery phase as well.

ABA, a plant hormone, participates in drought perception and signal transduction pathways [26,27]. Drought-induced ABA biosynthesis initiates ABA relocation and accumulation in guard cells, causing a loss of guard-cell turgor pressure which results in stomatal closure [28]. Since drought-tolerance responses include ABA-dependent and ABA-independent pathways, most drought-inducible genes in ABA-dependent pathway are also induced by exogenous ABA treatment [27,29]. The application of exogenous ABA can decrease plant transpiration rate [30], and inhibit seed germination [31] and plant growth under non-stress conditions [32]. As the MEP pathway is responsible for the generation of precursors for both ABA and isoprene biosynthesis, a direct linear correlation between isoprene emission and leaf ABA content was observed in both *Phragmites australis* and *Populus alba* at certain range of stomatal conductance [33]. Additionally, the decrease of isoprene emission was accompanied by the reduction of foliar ABA concentration when the MEP pathway was inhibited [33]. However, this correlation was not maintained under drought stress. When PaIspS (isoprene synthase from *Populus alba*) transgenic tobacco plants were subjected to mild drought stress, the dramatic decrease of isoprene emission did not affect the foliar ABA concentration and stomatal conductance at the whole-leaf level [24]. In turn, under severe drought stress, the isoprene emission of PaIspS transgenic tobacco plants was reduced, but the foliar ABA concentration was dramatically increased and unexpectedly higher stomatal conductance in PaIspS transgenic plants compared with non-emitter was detected [25]. According to these results, which are in contrast to current knowledge about the correlation between ABA level and stomatal conductance, it was suggested that the tobacco plants changed their water-management strategy from isohydric to anisohydric under drought stress.

Isohydric and anisohydric strategies are two water-management strategies in plants under water-limited conditions [34], although the distinctions between them are often not easy to identify precisely in practice [35,36]. Even plants in a given species could adjust their behavior types in different experimental conditions [37,38,39]. In general, plants with anisohydric behavior are more drought-tolerant [40,41,42], and faster to recover during re-watering than isohydric plants [35]. The high photosynthesis rate maintained by anisohydric plants under normal and mild drought conditions creates a growth advantage in uncertain and unpredictable environments (reviewed in Reference [43]). Additionally, the switch from isohydric to anisohydric strategies by gene transformation was reported to significantly increase fruit yield [42]. Probably due to ABA’s multiple roles in the regulation of water-use efficiency [43], the level of anisohydric behavior and isohydric behavior has been reported to strongly depend on abscisic acid (ABA) production and sensitivity [37,43,44], as anisohydric plants display low sensitivity and isohydric plants display hypersensitivity to ABA.

Recently, the first *IspS* gene was isolated from a monocot species, *Arundo donax* L., belonging to Poaceae family (*AdoIspS*) [45], which is an excellent energy crop and biomass feedstock [46,47] with a high growth rate and resistance to biotic and abiotic stresses [48]. Li and colleagues reported that the *AdoIspS* gene was upregulated at different time points when subjected to heat and osmotic stresses [45], andadditionally, a recent study showed that there were differences upon isoprene-emission rate identified between two *Arundo donax* ecotypes, and the higher isoprene emitter exhibited greater drought tolerance and faster recovery [49]. Taken together, these observations imply that isoprene emission in *A. donax* might play a positive role in plant adaptation to abiotic stresses in general. To date, intensive studies have been focused mainly on the comparison of physiological responses under different abiotic stresses between natural isoprene emitters and non-emitters, transgenic isoprene emitters, and corresponding control plants [50], but limited information is available upon physiological responses at different developmental stages under abiotic stress treatment, and the correlation between the physiological responses and molecular reactions. Thus, in this study we aimed to evaluate (1) the water-deficiency responses of AdoIspS transgenic plants compared with Col-0 at different developmental stages, and consequence of physiological responses derived from molecular reactions; (2) short-term responses of these genotypes at the young seedling stage and long-term responses of plant fitness under heat stress between these two genotypes; and (3) the consequent effects of the transformed *AdoIspS* gene on plant development and yields.

## 2. Results

### 2.1. Enhanced Tolerance of AdoIspS Transgenic Arabidopsis Plants to Exogenous ABA Treatment

**Figure 1:**
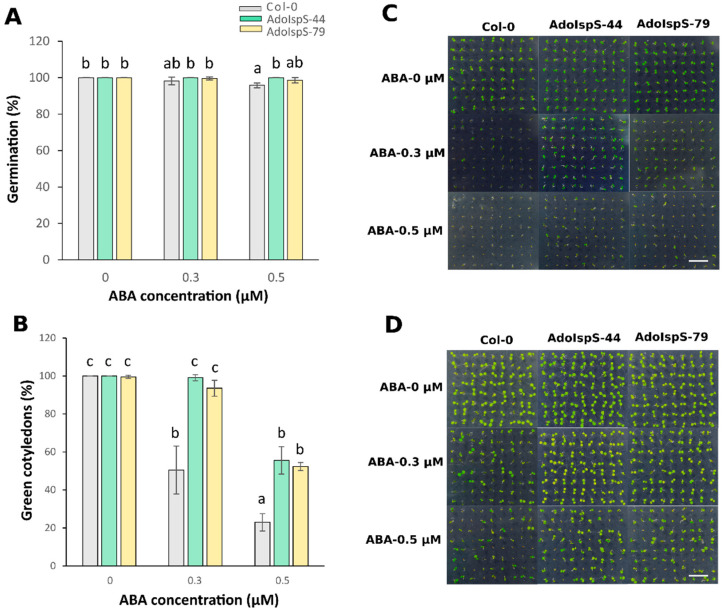
Effect of ABA on germination of transgenic lines overexpressing the *IspS* gene. (**A**) Germination rates of Col-0 and transgenic plants treated with different concentrations of ABA (0 µM, 0.3 µM, 0.5 µM) for 3 days. (**B**) Greening of cotyledons of Col-0 and transgenic plants exposed to different concentrations of ABA (0 µM, 0.3 µM, 0.5 µM) for 5 days. Histogram bars marked with the same letter do not significantly differ from each other (Tukey–Kramer test, *p* > 0.05). Error bars represent the SD of the means. In (**C**,**D**), representative pictures of germinating seedlings exposed to different concentrations of ABA at 3 days and 5 days after sowing are shown. Scale bar = 1 cm.

Expression of the *AdoIspS* transgene and isoprene emission from T3 homozygous transgenic lines AdoIspS-44 and AdoIspS-79 are shown in Supplementary Figures S1 and S2, respectively. The results showed that the two transgenic lines had comparable expression and emission levels, and that they were not subject to silencing. A previous study showed that *AdoIspS* was upregulated upon osmotic stress in *A. donax* [45]. It is well-known that ABA is a key regulator of abiotic stress responses [51]. To evaluate the response of AdoIspS transgenic plants to ABA treatment, seed germination analyses were carried out on half-strength MS medium supplemented with different concentrations of ABA. A two-factor ANOVA (3 × 3) was conducted to examine the effect of genotype and ABA concentration on germination rate. The results indicated a significant main effect for ABA treatment, F (2, 18) = 7.96, *p* = 0.0034, and a significant main effect for genotype, F (2, 18) = 9.71, *p* = 0.0014. There was a statistically significant interaction between the effects of genotype and ABA concentration on germination rate, F (4, 18) = 3.4825, *p* = 0.028. The germination rate of AdoIspS seeds was not consistently higher than that of Col-0 seeds treated with 0.5 μM ABA after 3 days of growth, and no difference could be observed after 5 days (Figure 1A). The rate of green cotyledon formation was significantly higher in both AdoIspS transgenic lines than that in Col-0 at both ABA concentrations (Figure 1B–D).

**Figure 2:**
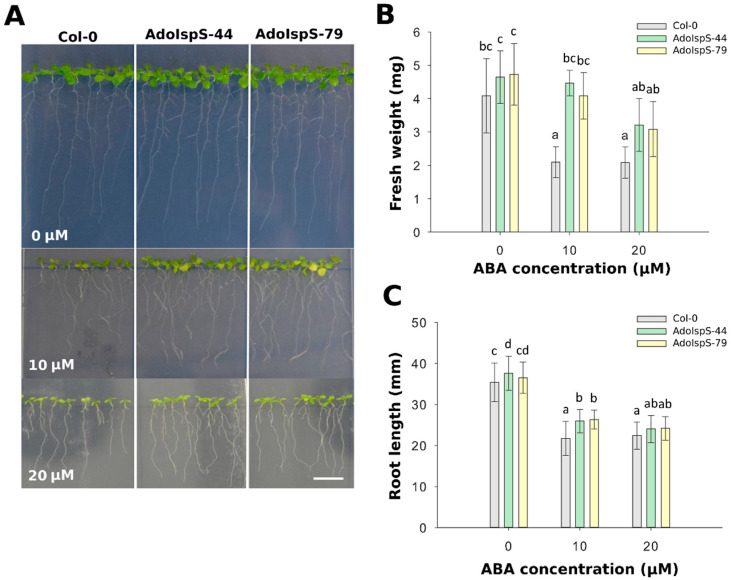
Effect of ABA on fresh weight and root elongation of transgenic lines overexpressing the *IspS* gene. (**A**) Representative pictures of Col-0 and transgenic seedlings grown on vertical plates and treated for 1 week with 0 µm, 10 µm, or 20 µM ABA. Scale bar = 1 cm. (**B**) Total fresh weight and (**C**) root length of plants treated with different amounts of ABA. Histogram bars marked with the same letter do not significantly differ from each other (Tukey–Kramer test, *p* > 0.05). Error bars represent the SD of the means.

To examine whether the decreased sensitivity of AdoIspS transgenic plants to exogenous ABA treatment during germination would be also maintained at the post-germination stage, 4 day old seedlings growing in half-strength MS medium were transferred either to the same medium or to medium supplemented with 10 or 20 µM ABA and grown for an additional 7 days. A two-factor ANOVA (3 × 3) was conducted to examine the effect of genotype and ABA concentration on either fresh weight or root length. The results for fresh weight indicated a significant main effect for ABA treatment, F(2, 45) = 23.01, *p* = 1.31 × 10^−7^, and a significant main effect for genotype, F(2, 45) = 17.58, *p* = 2.29 × 10^−6^. There was no statistically significant interaction between the effects of genotype and ABA concentration on fresh weight, F (4, 45) = 2.44, *p* = 0.061. The results for root length indicated a significant main effect for ABA treatment, F(2, 343) = 463.48, *p* <2. 2 × 10^−16^, and a significant main effect for genotype, F(2, 343) = 20.92, *p* = 2.7 × 10^−9^. There was statistically significant interaction between the effects of genotype and ABA concentration on root length, F (4, 343) = 4.26, *p* = 0.0022. Compared with Col-0, at 10 µM ABA concentration, the primary roots were significantly longer (Figure 2A), and the fresh weight was significantly higher than the Col-0 plants (Figure 2B,C). These results indicated that the overexpression of *AdoIspS* gene not only promoted the root growth of transgenic plants, but also the growth of aerial parts, thus relieving the growth inhibition associated with exogenous ABA treatment.

**Figure 3:**
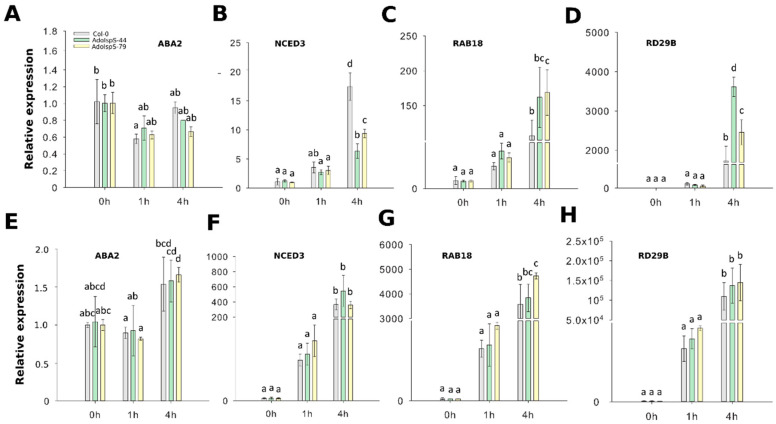
Expression of ABA biosynthetic and signaling genes in response to ABA and PEG stresses. (**A**–**D**) qRT–PCR analysis of ABA biosynthetic and signaling genes in the leaf tissue of IspS transgenic plants at different time-points after ABA treatment. (**E-H**) qRT–PCR analysis of ABA biosynthetic genes and signaling genes in the roots of IspS transgenic plants after ABA treatment. Histogram bars marked with the same letter do not significantly differ from each other (Tukey–Kramer test, *p* > 0.05). Error bars represent the SD of the means.

To evaluate whether ABA biosynthesis and signaling pathways were affected or not in transgenic plants overexpressing *AdoIspS*, four genes were selected for qRT-PCR analyses, namely *NCED3* and *ABA2* (related to ABA biosynthesis), and *RAB18* and *RD29B* (related to ABA signaling). Two-factor ANOVA (3 × 3) analyses were conducted to examine the effect of genotype and time on relative gene expression in shoot and root. The results for the two-way ANOVAs (Supplementary Table S1) indicated a significant main effect for time in all tests, F(2, 18) > 15.61, *p* < 0.0001. A significant main effect for genotype, F(2, 18) > 26.572, *p* < 4.25 × 10^−6^, was found in shoots for the effect of genotypes on relative expression levels of genes *NCED3* (downregulation in transgenic lines compared to WT) and *RD29B* (upregulation). There was a statistically significant interaction between the effects of genotype and time on expression levels for genes *NCED3*, *RAB18*, and *RD29B*, F (4, 18) > 2.95, *p* < 0.05 (details in Supplementary Table S1). In the case of *ABA2* expression, there were no significant differences detected in both shoots and roots between Col-0 and transgenic lines (Figure 3A,E). The transcripts of *NCED3* were significantly reduced in aerial parts of transgenic plants compared with those of Col-0 (Figure 3B), but in roots the expression levels of this gene were not different between AdoIspS transgenic plants and Col-0 (Figure 3F).

ABA-responsive marker genes *RAB18* and *RD29B* from both leaves and roots in both transgenic lines were highly expressed after exogenous ABA treatment (Figure 3C,D,G,H).

In summary, in leaves, AdoIspS plants showed upregulation of expression levels of two ABA-induced genes, and of expression of one ABA biosynthesis gene, but this pattern was not apparent in roots.

### 2.2. Enhanced Tolerance of AdoIspS Transgenic Arabidopsis Plants to Dehydration Stresses

**Figure 4:**
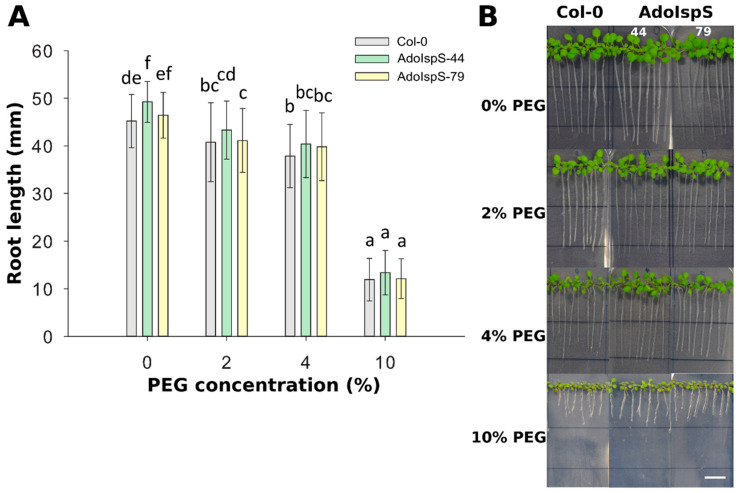
Effect of PEG-induced osmotic and water-limitation stress on root elongation. (**A**) Root length of Col-0 and transgenic plants exposed to various concentration of PEG 6000. Histogram bars marked with the same letter do not significantly differ from each other (Tukey–Kramer test, *p* > 0.05). Line bars represent the SD of the means. (**B**) Representative pictures of root growth of Col-0 and transgenic plants exposed to various concentrations of PEG 6000. Scale bar = 1 cm.

In order to examine the responses of AdoIspS transgenic plants to osmotic stress, 4 day old seedlings from Col-0 and AdoIspS transgenic plants were grown in nutritional solution supplemented with different concentrations of PEG 6000. After 7 days of treatment, a strong reduction of plant growth was observed for all genotypes (Figure 4). Two-factor ANOVA (4 × 3) analysis was conducted to examine the effect of genotype and PEG concentration on root length. The results for the two-way ANOVA indicated a significant main effect for PEG concentration, F(3, 888) = 1528.84, *p* < 2.2 × 10^−16^, and for genotype, F(2, 888) = 14.97, *p* = 4.036 × 10^−7^. There was no statistically significant interaction between the effects of genotype and PEG concentration on root length, F (6, 888) = 1.18, *p* = 0.32. In none of the conditions tested did AdoIspS transgenic lines show consistent differences in tolerance to osmotic stress compared with Col-0, as assessed by root length.

**Figure 5:**
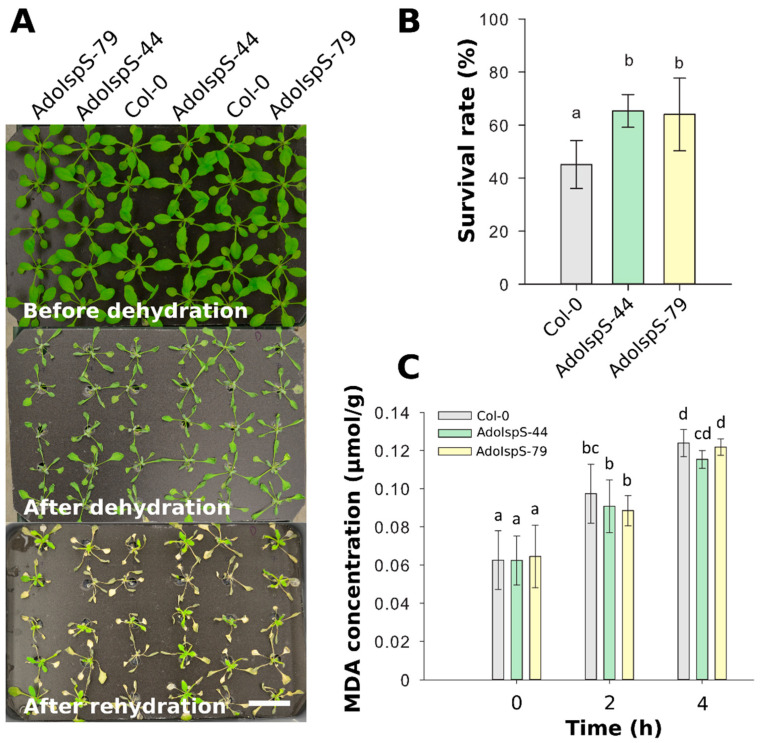
Water-stress tolerance of Arabidopsis plants overexpressing *AdoIspS*. (**A**) Drought resistance of Col-0 and AdoIspS transgenic plants. Plants were directly exposed to air for 24 h to induce dehydration. After rehydration for 48 h, the representative images were taken (Scale bar = 3 cm), and (**B**) the percentage of plants that survived was determined. (**C**) Lipid peroxidation was assessed by MDA accumulation at different time points. Histogram bars marked with the same letter do not significantly differ from each other (Tukey–Kramer test, *p* > 0.05). Line bars represent the SD of the means.

To further evaluate the dehydration response at later developmental stages, the survival rates of 18 day old plants growing in nutrition solution were recorded after 1 day of dehydration and 2 days of recovery. One-way ANOVA was conducted to examine the effect of genotype on survival rate. The results for the analysis indicated a significant effect of genotype on survival rate, F(2, 14) = 6.26, *p* = 0.013. Compared with Col-0, both AdoIspS transgenic lines showed significantly higher survival rates (Figure 5A,B). Furthermore, to examine the oxidative stress caused by dehydration, plant lipid peroxidation was analyzed by assessing the accumulation of malondialdehyde (MDA). A two-factor ANOVA (3 × 3) was conducted to examine the effects of genotype and time on MDA concentration. The results indicated a significant main effect for time, F(2, 54) = 123.18, *p* < 2 × 10^−16^ but no significant main effect for genotype, F(2, 54) = 0.98, *p* = 0.38. There was no statistically significant interaction between the effects of genotype and time on MDA concentration, F (4, 54) = 0.5742, *p* = 0.6825. MDA content in both Col-0 and AdoIspS plants was significantly increased after dehydration treatment. No difference in MDA accumulation was found in transgenic lines compared to Col-0 (Figure 5C).

**Figure 6:**
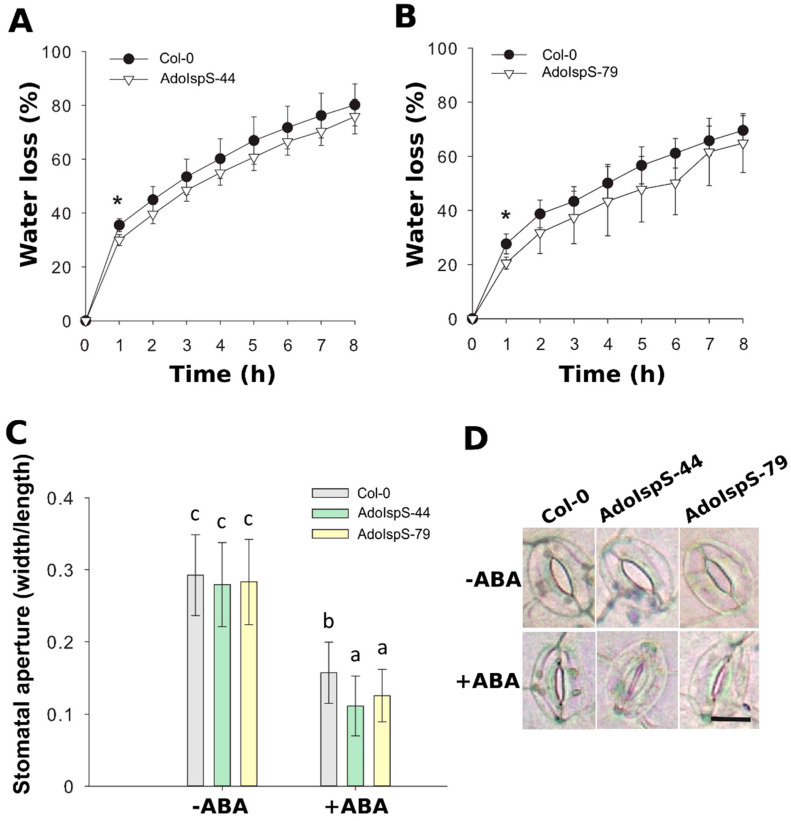
Water loss and stomatal aperture of Arabidopsis plants overexpressing IspS. (**A**,**B**) Water loss from the leaves of Col-0 and transgenic plants at various time-points after leaf detachment. Asterisks indicate significant differences between Col-0 and the transgenic lines (*t*-test, *p* < 0.05). Line bars report the standard deviation of the mean. (**C**) Stomatal apertures in Col-0 and IspS transgenic plants treated with ABA. Histogram bars marked with the same letter do not significantly differ from each other (Tukey–Kramer test, *p* > 0.05). Line bars represent the SD of the means. (**D**) Representative images of stomata from the different genotypes before and after ABA treatment. Scale bar = 10 µm.

In addition, the detached rosette leaves from 3 week old plants growing in soil were used for a transpiration rate assay. The water loss rate was significantly lower in both AdoIspS transgenic lines than in Col-0 in the first hour, while the rate increased in a similar manner later on (Figure 6A,B). In order to dissect the cause of the lower transpiration rate of AdoIspS transgenic plants, stomatal apertures were measured of leaves of Col-0 and AdoIspS plants treated with ABA. A two-factor ANOVA (2 × 3) was conducted to examine the effect of genotype and ABA on stomatal aperture. The results indicated a significant main effect for ABA concentration, F(1, 1020) = 903.95, *p* < 2.2 × 10^−16^, and a significant main effect for genotype, F(2, 1020) = 11.75, *p* = 8.97 × 10^−6^. There was a statistically significant interaction between the effects of genotype and ABA concentration on stomatal aperture, F (2, 1020) = 5.26, *p* = 0.0053. In the absence of ABA, no obvious difference was detected between Col-0 and AdoIspS plants upon stomatal aperture. However, after incubation with ABA, AdoIspS plants exhibited a significantly lower width:length ratio than Col-0. Thus, AdoIspS plants showed enhanced ABA-induced stomatal closure (Figure 6C,D).

**Figure 7:**
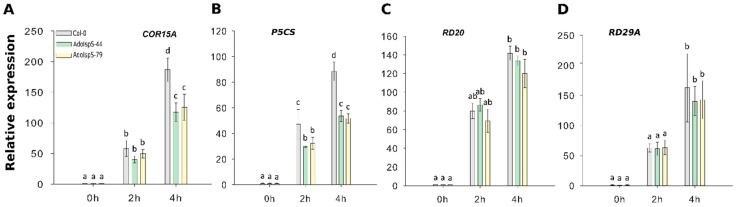
Expression of drought-responsive genes following dehydration stress. (**A**–**D**) qRT–PCR analysis of drought-inducible genes in IspS transgenic plants in response to dehydration at different time-points. Histogram bars marked with the same letter do not significantly differ from each other (Tukey–Kramer test, *p* > 0.05). Line bars represent the SD of the means.

To evaluate in more depth the dehydration response at the molecular level, qRT-PCR was carried out using the stress-related marker genes *COR15A*, *P5CS*, *RD20*, and *RD29A*. Two-factor ANOVA (3 × 3) analyses were conducted to examine the effect of genotype and time on relative gene expression. The results for the two-way ANOVAs (Supplementary Table S2) indicated a significant main effect for time in all tests, F(2, 18) > 34.69, *p* < 6.68 × 10^−7^. A significant main effect for genotype, F(2, 18) > 14.86, *p* < 0.00015, was found for the effect of genotype on relative expression levels of genes *COR15A* and *P5CS* (both downregulated in transgenic lines compared to WT). There was a statistically significant interaction between the effects of genotype and time on expression levels for genes *COR15A* and of *P5CS*, F (4, 18) > 8.5, *p* < 0.0005 (details in Supplementary Table S2). Dehydration treatment caused a large increase in gene expression levels relative to non-treatment. Among these four genes, the expression levels of *COR15A* and *P5CS* were significantly elevated in Col-0 compared with AdoIspS plants (Figure 7A,B); no differences among genotypes were detected in *RD20* or *RD29A* expression levels in response to dehydration stress (Figure 7C,D). These results may indicate that AdoIspS plants maintained a higher water potential.

### 2.3. Enhanced Tolerance of AdoIspS Transgenic Arabidopsis Plants to Heat

**Figure 8:**
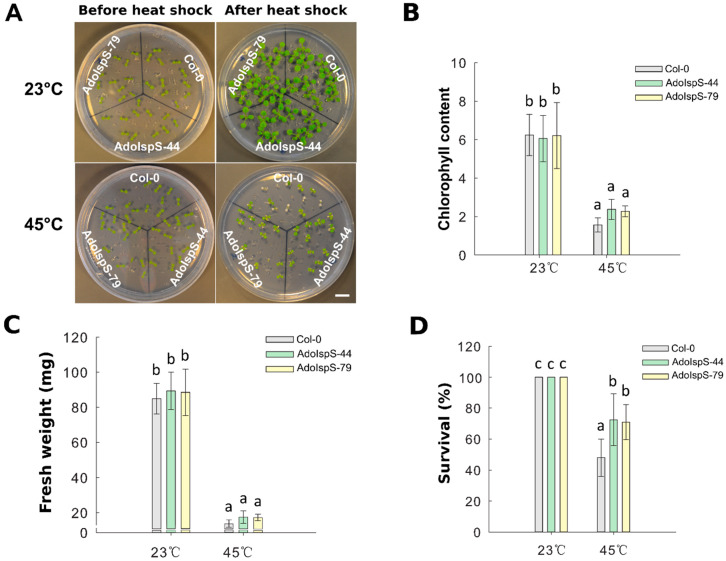
Thermal tolerance of Col-0 and IspS seedlings. Seven day old seedlings were exposed to heat shock. After 7 days recovery, representative images were taken (Scale bar = 1 cm) (**A**), and the chlorophyll content (**B**), fresh weight (**C**), and survival rate (**D**) were measured in each line. Histogram bars marked with the same letter do not significantly differ from each other (Tukey–Kramer test, *p* > 0.05). Line bars represent the SD of the means.

A previous study demonstrated that *AdoIspS* expression was significantly upregulated upon heat treatment in *A. donax* [45]; in addition, the *IspS* gene from poplar could increase tolerance of transgenic Arabidopsis to heat stress at a later developmental stage [23]. Until now, limited studies examining the early developmental stage under heat treatment were available. Therefore, the resistance to heat shock of Col-0 and AdoIspS transgenic plants was examined to evaluate whether AdoIspS transgenic plants also exhibited an enhanced thermo-tolerance at early developmental stages. Seven day old seedlings were treated at 45 °C for one hour. Two-factor ANOVAs (2 × 3) were conducted to examine the effect of genotype and temperature on chlorophyll content, fresh weight, and survival rate. A significant main effect for temperature only was found for chlorophyll content and fresh weight, with, respectively, F(1, 30) = 217.96, *p* = 2.673 × 10^−15^ and F(1, 30) = 716.37, *p* < 2 × 10^−16^. In the case of survival rate, the two-factor ANOVA results indicated significant main effects for both temperature and genotype, with, respectively, F(1, 30) = 127.89, *p* = 2.42 × 10^−12^ and F(2, 30) = 6.13, *p* = 0.0059. There was a statistically significant interaction between the effects of genotype and temperature on survival rate, F (2, 30) = 6.13, *p* = 0.0059. After a recovery phase, significantly more AdoIspS than Col-0 seedlings survived, although the chlorophyll content and fresh weights of both AdoIspS lines did not differ from WT. Compared with Col-0, recovered AdoIspS seedlings showed less necrosis (Figure 8).

**Figure 9:**
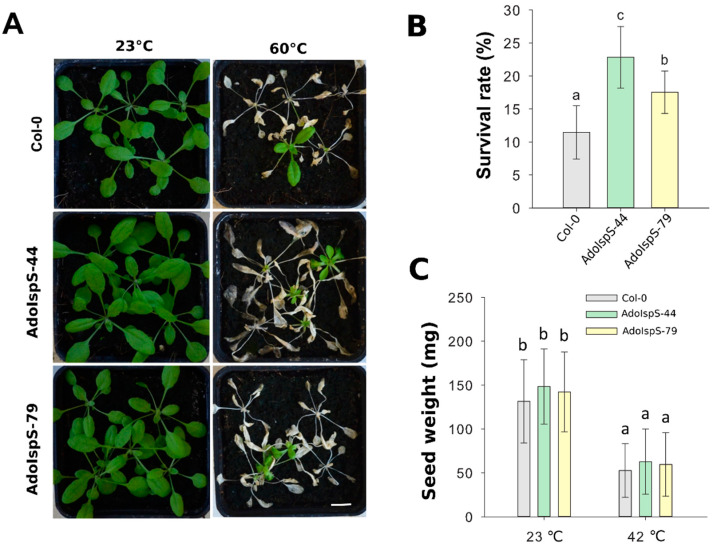
Thermal tolerance of Col-0 and IspS plants grown hydroponically. (**A**) Thermal tolerance of Col-0 and IspS transgenic plants grown in hydroponic medium. Three week old plants were treated with heat shock, representative images were taken (Scale bar = 1 cm) (**A**), and the percentages of surviving plants (**B**) were measured after 1 week of recovery. (**C**) Total weight of seeds produced per plant in Col-0 and IspS lines under normal growth and heat-shock condition. Histogram bars marked with the same letter do not significantly differ from each other (Tukey–Kramer test, *p* > 0.05). Line bars represent the SD of the means.

In addition, 3 week old plants growing in pots were treated at 60 °C for 2.5 h under dim light conditions. One-way ANOVA was conducted to examine the effect of genotype on survival rate. The results for the analysis indicated a significant effect of genotype on survival rate, F(2, 14) = 15.43, *p* = 0.00048. After 7 days of recovery, the survival rates of both AdoIspS transgenic lines were significantly higher than that of Col-0 (Figure 9A,B).

### 2.4. Alteration of Inflorescence Architecture of AdoIspS Transgenic Arabidopsis Plants

Previous studies showed that the leaves of transgenic plants overexpressing *PcIspS* or *PaIspS* in Arabidopsis grew bigger compared to those of wild-type plants in normal growth conditions, but no differences were observed in siliques or seeds [22,23]. Based on these previous analyses, further investigation on the phenotypic variation between wild-type and AdoIspS transgenic plants was performed. No differences were detected in the total weight of seeds produced per plant (Figure 9C) and seed set per silique (Supplementary Figure S3). Unexpectedly, compared with Col-0, AdoIspS transgenic plants produced more lateral and axillary branches, which led to an increase of the total number of siliques produced per primary shoot and per plant by AdoIspS transgenic plants (Supplementary Figure S3). These changes in inflorescence architecture had not been previously reported.

## 3. Discussion

Our current knowledge of the role played by isoprene emission in abiotic-stress tolerance largely stems from a conspicuous body of functional studies on the physiology of transgenic tobacco or Arabidopsis plants overexpressing *IspS* genes from various species [10,22,23,45,52,53]. To date, by contrast, the molecular mechanisms underlying the widely characterized physiological responses of plants to isoprene are still poorly understood. In this study, we combined physiological and molecular approaches to investigate in greater depth such mechanisms in relation to major types of abiotic stress, like water deficiency, excess of ABA, and high temperature.

The general characterization of our transgenic lines via stress-tolerance tests analogous to those conducted in previous publications confirmed that they behaved as other isoprene-emitting (IE) Arabidopsis lines. Survival of desiccation and survival of heat stress (Figure 4, Figure 5, Figure 8 and Figure 9) were, in fact, all in agreement with previous observations, suggesting that isoprene emission confers enhanced water-stress tolerance and heat-stress tolerance in transgenic models engineered to emit isoprene [23,25]. Previous studies, however, failed to identify a link of causal relationships between isoprene and ABA in the short term, despite the expectation that the diversion of DMADP from the MEP pathway might reduce ABA biosynthesis [25]. Whole-leaf ABA levels were found not to correlate to stomatal conductance, as both increased in IE plants compared to WT under the same levels of dehydration stress [25]; the studies referred mainly to medium- to long-term stress responses. Our results provide direct evidence of the effect that isoprene emission has on ABA-driven processes like stomatal closure, seedling greening, root elongation, and gene expression. The reason for the lack of correlation between whole-leaf ABA levels and stomatal conductance observed in previous studies [25] may be related to the fact that the action exerted by isoprene is both temporally and spatially restricted. Concerning time, we found that the effect of isoprene emission on water usage was significant only within the first hour upon stress application, as demonstrated by the time course of early desiccation stages assessed in this work (Figure 6A,B). Such time-restricted water-conservative behavior of isoprene-emitters likely explains the previous observations that isoprene emission seems to have relevance in short-term water stress, while the trait may not be ecologically relevant for taxa subjected to prolonged and intense heat or water-deprivation stresses [5,24,25]. The spatial evidence comes from the link between the action of isoprene and stomatal guard cells. First of all, the stomatal aperture test demonstrated that stomata of isoprene-emitting plants were more responsive to ABA, i.e., they closed more, than WT (Figure 6C,D). According to the most widely accepted definition of the term [54], the transgenic emitters showed an anisohydric, water-conservative behavior. This was in contrast to what previously reported for transgenic tobacco subjected to long-term water stress [25]. More recently, isoprene emission has been demonstrated to have partly contrasting effects on Arabidopsis and tobacco, where the trait is associated with yield penalties [24,50,55], which could be caused by different water-usage behavior. The second piece of evidence that isoprene action is mediated by guard-cell specific action came from *RD29B*, which together with *RAB18* is an important component of the ABA signaling pathway specific to the stomata in the leaf [56,57]. *RD29B* was upregulated in IE plants compared to WT, consistently with its known inducibility in the presence of ABA [56,58,59,60,61]. Both of the genes were found to depend for activation on the HAB1-SWI3B complex (formed by the protein phosphatase type 2C HYPERSENSITIVE TO ABA1 and the chromatin remodeling factor SWITCH SUBUNIT 3B), while the expression of *RD29A* and *P5CS1* genes was HAB1–SWI3B-independent [58]. Thus, the differences in regulation observed in this study indicate the presence of fine-tuning regulatory mechanisms deserving further investigation. It is noteworthy that, at the same time, the ABA biosynthetic gene *NCED3* was downregulated, suggesting a concomitant reduction of ABA biosynthesis consequent to exogenous hormone administration (Figure 3A,B). While ABA’s positive feedback loop on its biosynthetic genes is well established [62], comparatively little is known on how such a self-reinforcing loop is stopped in order to avoid ABA overproduction. Repressors of both of the genes analyzed in this work have been identified [63], but whether their expression can be modulated by exogenous ABA has not been investigated. Possibly, due to the fact that leaf mesophyll cells are the primary site of ABA biosynthesis in leaf [64], its synthesis may have to be finely regulated to prevent undesired stomatal closure.

Interestingly, the physiological data showed variations in the opposite direction of ABA responsiveness in seedlings (decreased ABA sensitivity) as compared to stomata (increased sensitivity), suggesting tissue-specific variation of the effects that isoprene–ABA interaction has on the following abiotic stress responses. These results were in line with the recent findings that isoprene acts as a signaling molecule in plants [50,65]. Isoprene has been proposed to downregulate *MARD1* in Arabidopsis but not tobacco [50]. As with SWI3B, *MARD1* loss of function also produces the same lack of sensitivity to ABA of seedlings at the radicle stage [66]. Further analyses are required to test the possible involvement of MARD1 and SWI3B in this specific branch of isoprene signaling.

In addition to ABA-mediated responses in the short term, however, the analysis of drought-responsive genes indicated the involvement of an osmotic component to the water tolerance mediated by isoprene. Two of the water-stress-related genes known to respond to ABA (*RD20* and *RD29A* [67,68]), in fact, were not differentially regulated between IE and WT (Figure 7C,D). By contrast, *COR15A* and *P5CS* were both downregulated in IE lines compared to WT (Figure 7A,B). The function of the COR15A protein is to stabilize membrane structure by reducing the formation of lamella-to-hexagonal II phase [69] and to stabilize chloroplast membranes in vivo [70]. The expression of *COR15A* is mainly induced by salt and cold, two potent elicitors of osmotic stress [71]. Besides *COR15A*, the expression level of *P5CS* in AdoIspS lines was also significantly lower than that of wild-type plants. P5CS is a rate-limiting enzyme which is responsible for synthesizing proline under stress conditions [72,73]. Many plants accumulate proline to offset the cellular imbalances caused by various stresses, including drought and osmotic stress [74]. Taking these results together with the enhanced tolerance to PEG-mediated osmotic stress, we propose that isoprene emission under drought stress can prevent damage to membrane integrity in response to water-deficit-driven osmotic stress. In recent years, evidence that isoprene affects membrane properties has been reported [15,75,76,77]. However, these observations are at odds with the estimated amount of isoprene in membranes, which is too little to justify a bulk effect [78]. The results presented here show that isoprene may explain this apparent contradiction by strengthening membranes not by a direct effect, but through changes in the expression of genes that play important roles in membrane integrity. Further investigation will be required to confirm this observation and, in particular, to ascertain the role of chloroplasts in such a mechanism.

In conclusion, we found evidence that isoprene emission is involved in short-term regulation of water-use efficiency by enhancing ABA sensitivity of stomatal cells through upregulation of the key ABA-signaling gene *RD29B*. In addition, isoprene is involved in other ABA-related physiological responses like seedling greening and root elongation, but in these cases, isoprene action led to a decreased sensitivity to the hormone, rather than to an increase as observed in stomata. These results provide additional support to the increasingly recognized function of isoprene as a signaling molecule and suggest novel directions of investigation in this exciting new development in the biological functions of this important bVOC. Future research directions include the detailed investigation of the role of ABA-signaling in relation to isoprene emission. In particular, the use of functional genomics and genetics approaches could help to further our mechanistic insights into the isoprene-mediated regulation of hormonal control of plant development and abiotic-stress tolerance.

## 4. Materials and Methods

### 4.1. Plant Materials and Growth Conditions

Arabidopsis Col-0 wild-type and two AdoIspS transgenic homozygous lines (AdoIspS-44 and AdoIspS-79) overexpressing the *isoprene synthase* gene from *Arundo donax* (GenBank accession No. KX906604) were used [45,79] and further characterized here. The promoter used for overexpression was the strong CaMV 35S promoter. Seeds of Col-0 and transgenic lines were surface-sterilized and sown on Murashige-Skoog (MS) agar medium containing 1% sucrose. After stratification at 4 °C for three days, the plates were transferred to a growth chamber (KBF 720, Binder GmbH, Tuttlingen, Germany) set at 23 °C and 40–50% relative humidity with light intensity of 100–120 μmol m^−2^·s^−1^ under long-day conditions (16 h light/8 h dark). A few days after germination, the seedlings were transferred to pots filled with commercial soil and grown in the same condition as mentioned above. The protocol used for plants growing in hydroponics was the one described by Tocquin and colleagues [80] and the plants were maintained in a growth chamber under the environmental conditions mentioned above [79].

### 4.2. Plant Architecture and Seed Production

The total numbers of secondary stems and axillary branches were assessed using 8 week old plants. For seed-productivity analyses, the seeds were collected by using Arasystems (AraSystem 360 KIT; Beta Tech bvba, Gent, Belgium) from 12 week old plants in two different temperature settings, normal growth condition (23 °C) and normal growth condition with a heat shock (42 °C) applied for two hours (11:00 to 13:00) every day since plants were 3 weeks old.

### 4.3. Seed Germination Assay and Root Growth Measurement

For germination tests, 72 seeds of each genotype were surface-sterilized and sown on Petri dishes containing half-strength MS medium supplemented either with 0.3 μM or 0.5 μM abscisic acid (ABA) dissolved in ethanol, while an equal amount of absolute ethanol without ABA was added to untreated control plates. Germination rates, which were based on radicle protrusion, were recorded after 3, 5, and 7 days of growth, and the percentage of green cotyledons was calculated after 7 days of growth. Three biological replicates were performed for each experimental condition.

To compare the response to ABA treatment among different genotypes during the post-germination stage, the root length was measured. Four day old seedlings growing on half-strength MS agar medium were transferred into square Petri dishes containing fresh medium supplemented either with 10 μM or 20 μM ABA or an equal amount of 99% ethanol (mock), and grown for an additional 7 days. Afterwards, the root length of vertically grown seedlings was captured with a stereomicroscope (MZ75, Leica Microsystems Srl, Buccinasco (MI), Italy) equipped with a color-CCD camera (DFC 420C, Leica Microsystems Srl, Buccinasco (MI), Italy), and the fresh weight of each plant was measured. Images were analyzed with the Leica Application Suite 2.8.1 software. Six biological replicates were performed for this experiment.

For osmotic-stress treatment with polyethylene glycol (PEG) 6000, 4 day old plants were transferred to the same hydroponic solution containing various concentrations of PEG 6000. Plants were photographed and root lengths were measured after 7 days of treatment. The root length and fresh weight were measured as described previously. At least 15 plants of each genotype were measured per treatment and five biological replicates were conducted correspondingly.

### 4.4. Stomatal Aperture Assay and Water Loss Measurement

Stomatal closing assays were measured as described previously by Ren and colleagues, with slight modification [81]. Rosette leaves of 4 week old plants were detached and incubated in a stomatal opening solution containing 10 mM MES, 50 μM CaCl_2_, and 10 mM KCl (pH 6.15), and exposed under fluorescent light (150 μmol m^−2^ s^−1^) for 3 h. The buffer was then replaced with freshly made opening solution containing 10 μM ABA for stomatal closing. After 3 h of treatment with ABA, about 40 stomata of each genotype were observed randomly under a differential interference contrast (DIC) microscope (DM 2500, Leica Microsystems Srl, Buccinasco (MI), Italy) with Leica Application Suite LAS V3.7 software. Image J 1.50i software (http://imagej.nih.gov/ij) was used to measure the length and width of individual stomata. Each experiment was repeated five times.

The water loss rate was assayed as follows. For each genotype, 15 rosette leaves of equal size were detached from five different 3 week old plants at the same developmental stage, and weighed immediately at each indicated time-point with five replicates.

### 4.5. Dehydration Treatment and Lipid Peroxidation Quantification

18 day old Col-0 and AdoIspS transgenic plants growing hydroponically were lifted out of the solution and directly exposed to air for 24 h, after which plants were rehydrated by putting them back into the same solution to recover for 48 h. Plants with rehydrated primary shoots were counted as survival events. This experiment was performed in five replicates, with about 20 plants for each genotype in each replicate.

Lipid peroxidation was quantified by measuring malondialdehyde (MDA) accumulation, using the TBARS method [82] with a slight modification. First, 100 mg of frozen plant material was homogenized in 1 mL of 0.1% (*w*/*v*) trichloroacetic acid (TCA) solution. The homogenate was centrifuged at 13,000 *g* at 4 °C for 20 min. Next, 0.5 mL of the supernatant was mixed with 1.5 mL of freshly made 20% TCA solution containing 0.5% (*w*/*v*) thiobarbituate acid (TBA). The mixture was heated in a boiling water bath for 30 min and cooled down in ice-cold water. Samples were centrifuged again for 5 min at 10,000 *g*. The supernatant absorbance was measured with an Ultrospec 3100 proUV/Visible Spectrophotometer (GE healthcare) at 532 nm, deducting the value at 600 nm to correct for non-specific turbidity. The extinction coefficient 155 mM^−1^ cm^−1^ was used to calculate the content of MDA–TBA complex. Results were obtained from seven biological replicates for each genotype.

### 4.6. Heat Shock Treatment and Chlorophyll Content Analysis

The heat survival test for Col-0 and AdoIspS transgenic plants at different developmental stages was carried out as follows: 7 day old seedlings were incubated at 45 °C in a pre-heated chamber for 1 h in the dark, and brought back to a 23 °C growth chamber for recovery. The survival rate was recorded after a week of recovery. Plants with four green leaves were scored as survival events. Each plate contained about 14 plants for each genotype as one replicate, and six replicates in total were performed. In addition, 3 week old plants growing in pots were transferred to a 60 °C growth chamber under dim light conditions (5 μmol·m^−2^·s^−1^). After 2.5 h of treatment, plants were moved back to the normal growth condition to revive. The percentages of surviving plants were calculated after 7 days of recovery. Six replicates were tested and each replicate was performed with 20 plants.

Leaf chlorophyll content was measured by homogenizing plants of each genotype using a TissueLyser II in the presence of 80% (*v*/*v*) ice cold acetone. After centrifugation for 13 min at 4600 rpm, the supernatant was collected and the pellet was macerated with acetone again and centrifuged as before. The absorbance of combined supernatant was determined according to the method described previously [83]. The absorbance was measured using a spectrophotometer at 663 nm for chlorophyll a and 645 nm for chlorophyll b. The Arnon equation was used for calculation of chlorophyll a concentration, Chl a = 12.7 × OD_663_ − 2.69 × OD_645_, while for chlorophyll b the equation was Chl b = 22.9 × OD_645_ − 4.68 × OD_663_, and for total chlorophyll content was C= 20.2 × D_645_ + 8.02 × D_663_. Six independent biological replicates were performed for this analysis.

### 4.7. RNA Isolation and qRT-PCR Analysis under Dehydration and ABA Treatments

Total RNA was extracted from Col-0 and AdoIspS transgenic plants using the Trizol reagent (Invitrogen) and treated with DNase I (Sigma) to eliminate genomic DNA contamination. After RNA quantification using a spectrophotometer and integrity control on agarose gel, 1 µg of total RNA was reverse-transcribed using SuperScriptIII (Invitrogen) to synthesize the first strand of cDNA. Quantitative real-time PCR (qRT-PCR) was performed using Platinum SYBR Green qPCR SuperMix-UDG (Invitrogen) according to the manufacturer’s instructions in a Bio-Rad C1000 Thermal Cycler detection system programmed as in Reference [84]. In brief, the qPCR reaction was performed by adding 1 µL of 10-fold diluted cDNA (5 ng of starting RNA), 200 nM of each primer, 6.25 µL of Platinum^®^ SYBR^®^ Green qPCR SuperMix-UDG (Invitrogen) and H_2_O to reach a final volume of 12.5 µL. The qRT-PCR program was set as follows: 2 min at 50 °C, 2 min at 95 °C, 40 cycles of 15 s at 95 °C, and 30 s at 60 °C. The melting curves were recorded for every gene after Cycle 40 by constantly raising the temperature from 65 °C to 90 °C. A standard curve of qPCR reaction was generated from five points of a 4-fold dilution series. The slope (S) of the standard curve was used to calculate the amplification efficiency (E) of each primer pair as follows: E = 10 ^(−1/S)^. The qRT-PCR results are from at least three technical and three biological replicates. The relative transcription level of each gene was calculated with the 2^−ΔΔCT^ method and normalized to the mean of internal control *Actin* for the same sample. Primers used for this analysis are listed in Supplementary Table S3.

### 4.8. Statistical Analysis

Data with one independent variable (factor) were analyzed using one-way ANOVA analysis. Data with one independent variable (factor) were analyzed using two-way (two-factor) ANOVA analysis. Tukey’s multiple comparison and least-significant-difference (LSD) tests were used to identify significant differences. Differences were considered significant if *p* ≤ 0.05 in the two-sided test. Compact letter display was used to summarize the differences among means. All analyses were run in R version 4.0.0 (2020. 04.24 [85]) using the scripts provided in Reference [86].

## Supplementary Materials

Supplementary materials can be found at https://www.mdpi.com/1422-0067/21/12/4276/s1.

## Abbreviations

IspS	Isoprene synthase
ABA	Abscisic acid
ROS	Reactive oxygen species
DMADP	dimethylallyl diphosphate
MEP	methylerythritol 4-phosphate
PEG	Polyethylene glycol
MDA	malondialdehyde
qRT-PCR	Quantitative real time polymerase chain reaction
TCA	Trichloroacetic acid
TBA	Thiobarbituate acid
