# Supplementary material for: Overexpression of Isoprene Synthase Affects ABA- and Drought-Related Gene Expression and Enhances Tolerance to Abiotic Stress
Source: Int J Mol Sci. 2020 Jun 16;21(12):4276. doi: 10.3390/ijms21124276 (PMC7352718; doi:10.3390/ijms21124276)
Supplement: Supplementary file 1 [file ijms-21-04276-s001.pdf]

SUPPLEMENTARY LEGENDS

Supplementary Figure 1. Semi-quantitive PCR of *AdoIspS* expression in transgenic AdoIspS lines and Col-0 WT. *AtACT2*: *Arabidopsis thaliana Actin 2* housekeeping gene. In total, 26 cycles and 28 PCR amplification cycles were used for *AtACT2* and *AdoIspS*, respectively.

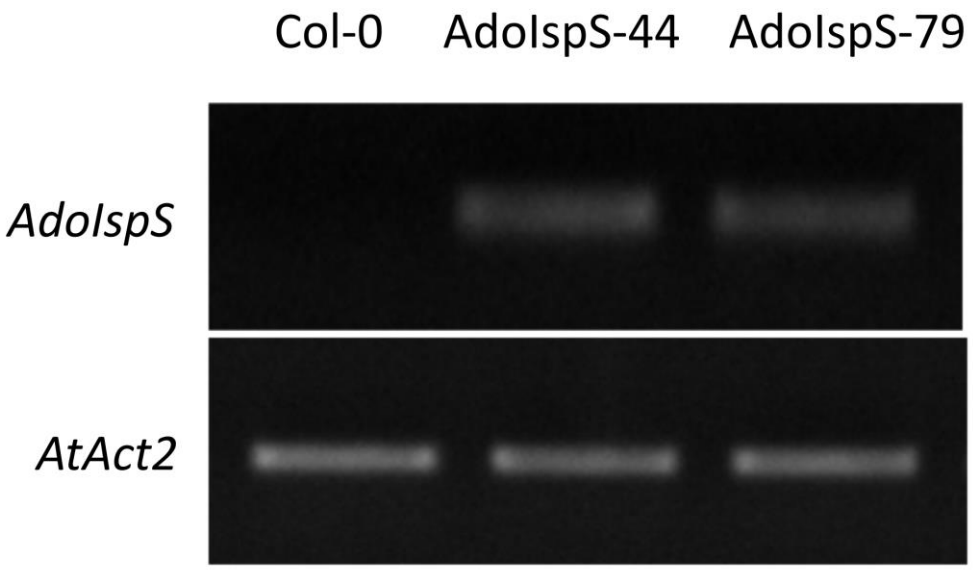

Supplementary Figure 2. Isoprene emission of transgenic AdoIspS lines and Col-0 WT. ppbv: parts per billion volume.

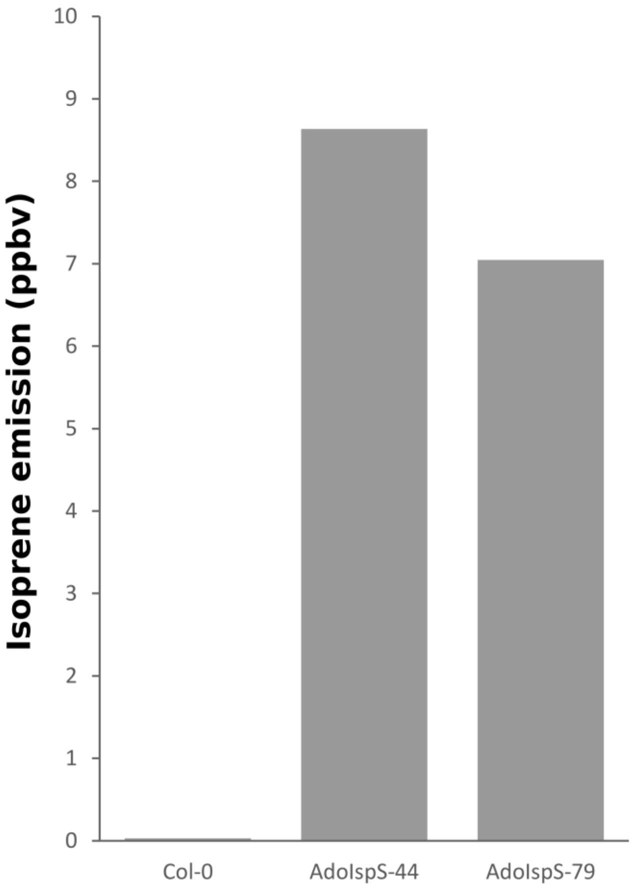

Supplementary Figure 3. Inflorescence architecture comparison between transgenic AdoIspS lines and Col-0 WT. The left y-axis refers to the number of secondary branches, axillary branches, siliques in the primary shoot and seeds per silique. Histogram bars marked with the same letter do not significantly differ from each other (Tukey–Kramer test, P>0.05). Error bars represent the SD of the means.

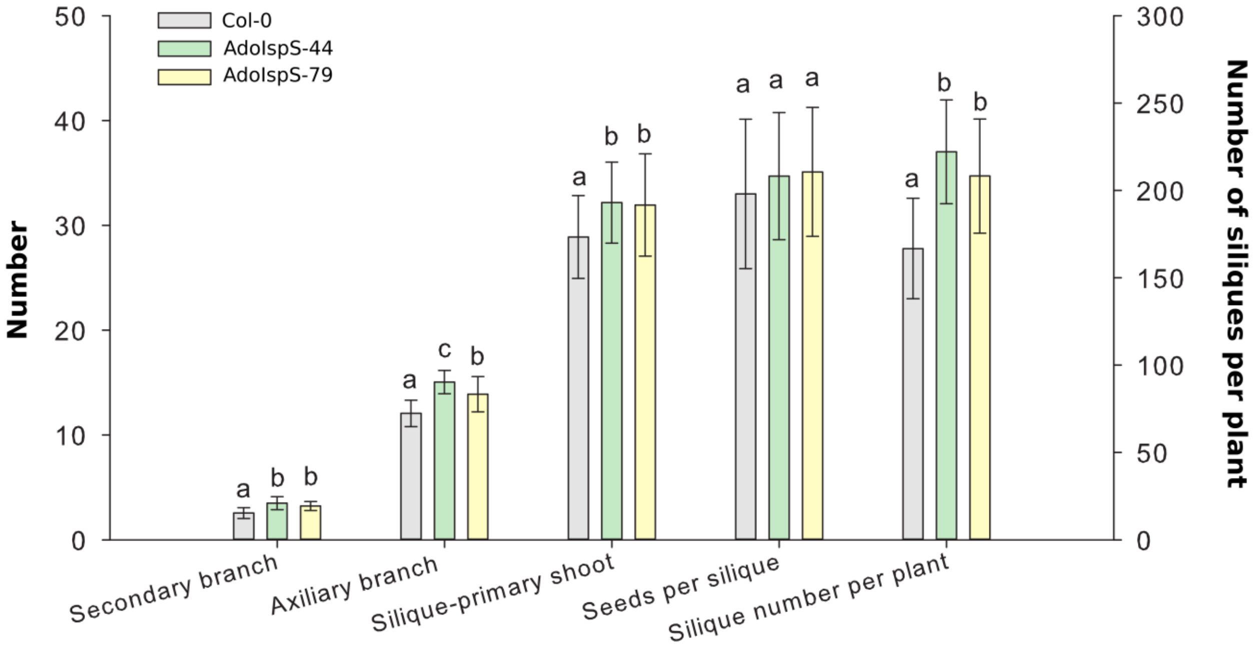

Table_S1_R1

qPCR OF ABA-RELATED GENES - TWO-WAY ANOVA TABLES SUMMARY

Fig. 3A – *ABA2* leaf

```
Response: Relative expression
                 Sum Sq Df F value   Pr(>F)    
Time         0.64500  2 15.6109 0.000117 ***
Genotype         0.01532  2  0.3708 0.695314    
Time:Genotype 0.03675  4  0.4447 0.774790    
Residuals       0.37186 18                     
---
Signif. codes:  0 '***' 0.001 '**' 0.01 '*' 0.05 '.' 0.1 ' ' 1
```

Fig. 3B – *NCED3* leaf

```
Response: Relative expression
                Sum Sq Df F value    Pr(>F)    
Time         499.34  2 233.971 1.313e-13 ***
Genotype          74.89  2  35.088 6.156e-07 ***
Time:Genotype 124.40  4  29.145 1.205e-07 ***
Residuals        19.21 18                      
---
Signif. codes:  0 '***' 0.001 '**' 0.01 '*' 0.05 '.' 0.1 ' ' 1
```

Fig. 3C – *RAB18* leaf

```
Response: Relative expression
                Sum Sq Df  F value    Pr(>F)    
Time         123327  2 161.6364 3.159e-12 ***
Genotype           2419  2   3.1705   0.06613 .  
Time:Genotype   4499  4   2.9480   0.04895 *  
Residuals          6867 18                       
```

Fig. 3D – *RD29B* leaf

```
Response: Relative expression
                  Sum Sq Df F value    Pr(>F)    
Time         39887710  2 589.714 < 2.2e-16 ***
Genotype          1797311  2  26.572 4.248e-06 ***
Time:Genotype  3654785  4  27.017 2.149e-07 ***
Residuals          608752 18                      
---
Signif. codes:  0 '***' 0.001 '**' 0.01 '*' 0.05 '.' 0.1 ' ' 1
```

Fig. 3E – *ABA2* root

```
Response: Relative expression
                 Sum Sq Df F value    Pr(>F)
```

```
Time         2.57328  2 25.9686 4.955e-06 ***
Genotype         0.00721  2  0.0728    0.9301
Time:Genotype 0.03692  4  0.1863    0.9425
Residuals      0.89183 18
```

Fig. 3F – *NCED3* root

```
Response: Relative expression
                 Sum Sq Df F value    Pr(>F)
Time         1020771  2 92.0017 3.542e-10 ***
Genotype           21738  2  1.9592    0.1699
Time:Genotype   44725  4  2.0155    0.1353
Residuals         99856 18
---
Signif. codes:  0 ‘***’ 0.001 ‘**’ 0.01 ‘*’ 0.05 ‘.’ 0.1 ‘ ’ 1
```

Fig. 3G – *RAB18* root

```
Response: Relative expression
                  Sum Sq Df  F value    Pr(>F)
Time         97561825  2 448.1078 4.446e-16 ***
Genotype           747532  2   3.4335   0.05456 .
Time:Genotype  1446898  4   3.3228   0.03326 *
Residuals        1959476 18
---
Signif. codes:  0 ‘***’ 0.001 ‘**’ 0.01 ‘*’ 0.05 ‘.’ 0.1 ‘ ’ 1
```

Fig. 3H – *RD29B* root

```
Response: Relative expression
                    Sum Sq Df F value    Pr(>F)
Time         1.0205e+11  2 85.9297 6.188e-10 ***
Genotype         6.8308e+08  2  0.5752    0.5726
Time:Genotype 1.3626e+09  4  0.5737    0.6852
Residuals       1.0688e+10 18
---
Signif. codes:  0 ‘***’ 0.001 ‘**’ 0.01 ‘*’ 0.05 ‘.’ 0.1 ‘ ’ 1
```

Table_S2_R1

qPCR OF DEHYDRATION-RELATED GENES - TWO-WAY ANOVA TABLES SUMMARY

Fig. 7A – *COR15A*

```
Response: Relative gene expression
                Sum Sq Df  F value    Pr(>F)    
FactorA          94363  2 330.9552 6.388e-15 ***
FactorB           4236  2  14.8557 0.0001548 ***
FactorA:FactorB   4849  4   8.5037 0.0004900 ***
Residuals         2566 18                       
---
Signif. codes:  0 ‘***’ 0.001 ‘**’ 0.01 ‘*’ 0.05 ‘.’ 0.1 ‘ ’ 1
```

Fig. 7B – *P5CS*

```
Response: DependentVariable
                 Sum Sq Df F value    Pr(>F)    
FactorA         18279.4  2 347.764 4.138e-15 ***
FactorB          1797.5  2  34.197 7.398e-07 ***
FactorA:FactorB  1288.2  4  12.254 5.518e-05 ***
Residuals         473.1 18                      
---
Signif. codes:  0 ‘***’ 0.001 ‘**’ 0.01 ‘*’ 0.05 ‘.’ 0.1 ‘ ’ 1
```

Fig. 7C – *RD20*

```
Response: DependentVariable
                Sum Sq Df F value    Pr(>F)    
FactorA          54777  2 34.6934 6.675e-07 ***
FactorB            655  2  0.4148    0.6666    
FactorA:FactorB    360  4  0.1140    0.9759    
Residuals        14210 18                      
---
Signif. codes:  0 ‘***’ 0.001 ‘**’ 0.01 ‘*’ 0.05 ‘.’ 0.1 ‘ ’ 1
```

Fig. 7D – *RD29A*

```
Response: DependentVariable
                Sum Sq Df F value    Pr(>F)    
FactorA          99070  2 86.6067 5.805e-10 ***
FactorB            314  2  0.2743    0.7632    
FactorA:FactorB    583  4  0.2549    0.9029    
Residuals        10295 18                      
---
Signif. codes:  0 ‘***’ 0.001 ‘**’ 0.01 ‘*’ 0.05 ‘.’ 0.1 ‘ ’ 1
```

Table_S3_R1

**Primer name**	**Sequence (5' - 3')**	**Tm ***	**PCR cycles**
ABA2_Fw	AAGCATGAAACATGCAGCTCG	60°C	40
ABA2_Rv	AAGAATGTGGACCAACGCCTC	60°C	40
NCED3_RT_Fw	ACATGGAAATCGGAGTTACAGATAG	60°C	40
NCED3_RT_Rv	AGAAACAACAAACAAGAAACAGAGC	60°C	40
RAB18_RT_Fw	GGAAGAAGGGAATAACACAAAAGAT	60°C	40
RAB18_RT_Rv	GCGTTACAAACCCTCATTATTTTTA	60°C	40
RD29B_RT_Fw	GTGAAGATGACTATCTCGGTGGTC	60°C	40
RD29B_RT_Rv	TACCAAGAGACTCAGCAATCTCTG	60°C	40
COR15A_RT_Fw	GATACATTGGGTAAAGAAGCTGAGA	60°C	40
COR15A_RT_Rv	ACATGAAGAGAGAGGATATGGATCA	60°C	40
RD20_RT_Fw	GCTTTCAAACAAAGTTGAATGGATAC	60°C	40
RD20_RT_Rv	TAGTCTTGTTTGCGAGAATTGGCC	60°C	40
RD29A_RT_Fw	GGAAGTGAAAGGAGGAGGAGGAA	60°C	40
RD29A_RT_Rv	CACCACCAAACCAGCCAGATG	60°C	40
AtActII_RT_Fw	GCACCCTGTTCTTCTTACC	60°C	40
AtActII_RT_Rv	AACCCTCGTAGATTGGCACA	60°C	40

* Annealing temperature
